# Assessing the tourism impacts of urban marathon events in central China's historic cities: a residents’ SEM analysis

**DOI:** 10.3389/fspor.2025.1720413

**Published:** 2025-12-17

**Authors:** LingLing Zhang, JinBai Liu, XiuMei Qiao, Denise Koh Choon Lian

**Affiliations:** 1 Faculty of Sports Management, Universiti Kebangsaan Malaysia, Kuala Lumpur, Malaysia; 2 Physical Education College, Anyang Normal University, Anyang, China

**Keywords:** city marathons, tourism, economic impacts, urban image, social exchange theory, theory of reasoned action

## Abstract

As an emerging form of sports tourism, city marathons have become an important means to stimulate local economic growth and enhance urban image. However, empirical evidence on how such events influence tourism development in economically underdeveloped but culturally rich cities remains limited. Drawing on Social Exchange Theory (SET) and the Theory of Reasoned Action (TRA), this study constructs a structural equation model (SEM) to examine, from the residents’ perspective, how perceptions of tourism economic impacts (TEC), tourism image impacts (TIM), and tourism spatial impacts (TSP) affect residents’ attitudes toward the marathon (RAT) and their intention to support it (RIS). Using the Kaifeng City Marathon as a case study, the results show that all three perceived impacts significantly and positively influence residents’ attitudes, with tourism image perception having the strongest effect. Positive attitudes, in turn, significantly enhance residents’ support intention. Mediation analysis further reveals that attitude fully mediates the effects of economic and image perceptions, while partially mediating the spatial perception. These findings provide empirical evidence for how sports events can promote tourism development in economically underdeveloped small cities.

## 1 Introduction

Sports tourism has become one of the fastest-growing sectors in the global tourism industry, reflecting society's increasing awareness of health, active lifestyles, and experiential travel (1). Today, sports tourism is no longer a niche phenomenon but a significant form of tourism with both cultural and economic importance. It integrates sports participation, leisure, and international exchange, creating diverse opportunities for urban development and cultural interaction (2–4).

Recent studies have shown that mega-events such as the Olympic Games can stimulate tourism consumption, enhance city visibility, and generate positive impacts on multiple dimensions, including the local economy and urban environment (5, 6). Over the past few decades, many countries have developed strategic frameworks for hosting mega-events and have invested significant resources to attract them (7) A classic example is Barcelona, where the 1992 Olympic Games greatly boosted tourism development and reshaped the city's global image. Owing to its well-organized event planning and extensive media promotion, Barcelona has since become one of the world's most recognized tourist destinations. This phenomenon, often referred to as the “Barcelona Effect,” illustrates how mega sporting events can transform a city's reputation and long-term tourism trajectory (8).

A growing body of research has examined the tourism impacts of mega sporting events such as the Olympic Games and the FIFA World Cup from multiple perspectives (9–11). However, in practice, these large-scale events are typically infrequent and entail extremely high costs (12). Due to financial and infrastructural limitations, small and medium-sized cities often lack the capacity to host such mega events within a short time frame (13, 14). In contrast, city marathons, typically classified as major events due to their substantial participation scale and recurring structure, offer several advantages. First, they attract a broader participant base. Over the past two decades, marathons have become increasingly popular among amateur athletes and recreational runners due to their low entry barriers (15). This trend has brought sports events closer to the general public. Second, city marathons encourage frequent travel and intercity mobility. For instance, Martin and Hall (16) found that hotel bookings in New York surged significantly during the marathon period, indicating increased travel frequency among visitors. Third, city marathons are characterized by low investment, operational flexibility, and strong replicability, making them an effective vehicle for urban branding and destination marketing (17). Fourth, many cities have established fixed annual race schedules, and some even hold both spring and autumn editions, forming a stable and predictable hosting rhythm (18). The sustained growth of marathon events plays a vital role in promoting national fitness initiatives and advancing the sports industry. In response, the Chinese government has introduced the Marathon Sports Industry Development Plan (2022–2025) to guide the high-quality development of marathon events, stimulate sports consumption, and enhance economic vitality (19).

For these reasons, city marathons are more adaptable to small and medium-sized cities. Due to their economic conditions and spatial constraints, such cities often lack the capacity to acquire large-scale fixed assets within a short period (20). In this context, the light-asset operational model of city marathons aligns well with the fiscal limitations and spatial capacity of small and medium-sized cities (13). Moreover, through effective route planning, marathon events can connect major tourist attractions with commercial districts, thereby stimulating cultural and tourism consumption and enhancing urban visibility at relatively low costs. This linkage mechanism helps generate sustainable tourism vitality that can be transformed into long-term economic benefits (21).

In city marathons, local residents serve as critical stakeholders. They are not only direct participants in maintaining event order and creating the on-site experience but also act as “communicators” who shape post-event word-of-mouth and the host city's image (21). Whether residents support hosting the event largely depends on their evaluation of potential benefits and costs, which aligns with the explanation offered by Social Exchange Theory (SET) (22). Moreover, both the Theory of Reasoned Action (TRA) and the Theory of Planned Behavior (TPB) posit that attitudes are effective predictors of behavioral intention (23). When residents perceive the tourism impacts of the event positively, they are more likely to develop favorable attitudes, which in turn translate into stronger intentions to support future events. When residents perceive the tourism impacts of the event positively, they are more likely to develop favorable attitudes, which in turn translate into stronger intentions to support future events. Recent studies further indicate that such support is often expressed through concrete behaviors, including volunteering for event operations, assisting visiting athletes and tourists, participating in community-based service activities, and acting as everyday ambassadors who promote the host city and its tourism offerings to others (24–26). Therefore, examining this relationship from the residents' perspective provides valuable insights for promoting high-quality tourism development in economically underdeveloped small and medium-sized cities.

Within the event-impact literature, tourism-related impacts such as economic, image, and spatial changes are increasingly understood as specific manifestations of broader social impacts. Getz (27) conceptualizes social impacts as changes in residents' quality of life, community cohesion, social capital, pride, and shared identity that arise from hosting events (27), while Deery and Jago underline that community well-being, patterns of social exchange, and perceived social costs are central in shaping residents' attitudes toward sport and event tourism (28). From this perspective, the economic, image, and spatial tourism impacts examined in this study function as key pathways through which events shape broader social outcomes. Enhancements in local income, destination visibility, and urban spatial organization can strengthen civic pride, deepen social cohesion, and reinforce residents' sense of shared identity. In contrast, issues such as crowding, environmental pressure, and disruptions to daily life may weaken these social benefits and reduce overall community support. Research shows that residents' support hinges on the balance between perceived benefits and costs—economic and image improvements strengthen support, whereas negative experiences weaken it (29–31). Situating tourism-related impacts within this broader social impact framework therefore provides a richer conceptual basis for understanding residents' attitudes and their willingness to support recurring city marathons.

This study selected Kaifeng, a small city in central China, as the research sample. Kaifeng is highly representative within China's urban landscape because of its well-preserved historical heritage and rich cultural symbols. Although the city possesses abundant cultural resources, its economic development and the level of tourism marketization remain limited due to its lower position within the urban hierarchy. At the same time, its proximity to Zhengzhou, the provincial capital, provides access to additional visitor flows through regional sporting activities. Within this setting, the Kaifeng City Marathon serves as an appropriate case for analysis. Established in 2007 and held annually in spring, it is an international road race certified by the Chinese Athletics Association and jointly organized by national and local sports authorities as well as municipal governments. It offers full, half, and 10 km categories and attracts around 49,000 participants each year, making it the most influential recurring marathon event in Henan Province. Its scale, regularity, and cultural visibility illustrate how sports events can be integrated with urban tourism in small and medium-sized cities.

Therefore, examining the Kaifeng Marathon provides valuable insights into how residents' support can be transformed into tourism momentum that contributes to local economic vitality and enhances the city's image. Building on this context, This study aims to examine how residents' perceptions of the economic, image, and spatial impacts of a city marathon shape their attitudes and support for the event.

## 2 Literature review

### 2.1 Theoretical foundations

This study develops a conceptual model illustrating the impact pathways of city marathons on urban development, grounded in Social Exchange Theory (SET) and the Theory of Reasoned Action (TRA). SET provides a useful framework for analyzing residents' perceptions, attitudes, and behavioral intentions, explaining why individuals choose to engage in or withdraw from social exchanges (32). According to this theory, residents' attitudes toward hosting sports events depend on their evaluation of the perceived benefits and costs such events bring to the host destination (33). During the organization of urban sporting events, residents inevitably participate in various forms of social exchange—some may gain tangible or intangible benefits, while others may experience inconvenience or loss. The theory further posits that individuals assess these exchanges based on their cognitive appraisal of rewards and costs (34). Therefore, residents' perceptions of the tourism impacts of sports events influence their overall attitudes toward the events, which in turn shape their intention to support future hosting efforts.

In addition, the Theory of Reasoned Action (TRA) proposed by Ajzen and Fishbein provides a solid theoretical foundation for this study (35). The theory has been widely applied in the social sciences to predict and explain individual behavior (36). TRA is composed of key constructs such as beliefs, attitudes, intentions, and behavior, making it highly suitable for examining the relationship between residents' attitudes toward sports events and their future intention to support them. According to this theory, attitude represents a psychological tendency reflecting the degree to which individuals favor or disfavor a specific object, shaped by their beliefs and knowledge about it. Furthermore, TRA posits that an individual's attitude directly influences behavioral intention, which subsequently leads to actual behavior (37). Therefore, based on TRA, it can be inferred that residents with positive attitudes toward hosting city marathons are more likely to express stronger intentions to support future events.

### 2.2 Tourism impacts of sporting events

Extensive research has investigated the impacts of sporting events on host cities. Scholars generally agree that although such impacts are complex, they can be systematically analyzed through the Triple Bottom Line (TBL) framework, which divides them into economic, social, and environmental dimensions (38, 39). Within this framework, sporting events generate both tangible and intangible outcomes that differ in scope, duration, and intensity (40).

Beyond the overall urban impacts, many studies have specifically examined the influence of sporting events on urban tourism development, including promoting tourism-driven economic growth, positioning cities as tourism destinations, improving the tourism environment, enhancing city image, and shaping urban tourism spaces (41, 42). Huang et al. (43) summarized that the tourism impacts of sporting events mainly manifest in three domains. First, the economic dimension involves increased tourist arrivals, higher visitor spending, greater tourism revenues, and an optimized industrial structure.

Second, the image dimension includes stronger media exposure, improved city reputation, the construction or renewal of landmark attractions, and the enhancement of destination identity. Third, the spatial dimension refers to the reorganization of urban spaces through the transformation of venues into tourist attractions, the integration of event routes with tourism corridors, and the spatial clustering of tourism resources. Moreover, empirical evidence indicates that residents' attitudes toward sporting events are largely shaped by their perceptions of tourism impacts (44, 45). Overall, previous studies support a positive relationship between perceived tourism impacts and residents' supportive attitudes toward event hosting. Therefore, based on Social Exchange Theory (SET) and Theory of Reasoned Action (TRA), and drawing on existing empirical findings, this study proposes the following hypotheses:

H1: Perceived Tourism Economic Impacts (TEC) of city marathons positively influence residents’ attitudes toward the event.
H2: Perceived Tourism Image Impacts (TIM) of city marathons positively influence residents’ attitudes toward the event.
H3: Perceived Tourism Spatial Impacts (TSP) of city marathons positively influence residents’ attitudes toward the event.

Attitude represents an individual's evaluative judgment of value (46). According to the Social Exchange Theory (SET) and the Theory of Reasoned Action (TRA), local residents' attitudes toward sporting events are shaped by their perceptions of tourism impacts associated with such events (22). In turn, these attitudes influence their behavioral intentions to support future events, which may further translate into actual behaviors (47). Although numerous studies have examined the relationship between residents' perceptions of event impacts and their support for sporting events (48, 49), few have systematically explored the integrated relationships among perceived tourism impacts, residents' attitudes, and future support intentions. Prior research has shown that residents' attitudes can effectively predict their willingness to support events (50), and that attitudes may serve as a mediating variable linking perceived event impacts and behavioral support (51). Based on these theoretical and empirical foundations, this study proposes the following hypotheses:

H4: Residents’ attitudes toward the city marathon positively influence their intentions to support future events.
H5: Perceived economic impacts of the city marathon on tourism are positively associated with residents’ support intentions through the mediating effect of attitude.
H6: Perceived image impacts of the city marathon on tourism are positively associated with residents’ support intentions through the mediating effect of attitude.
H7: Perceived spatial impacts of the city marathon on tourism are positively associated with residents’ support intentions through the mediating effect of attitude.

## 3 Research methodology

Participation in this study was voluntary and anonymous. All respondents provided informed consent before completing the survey, and no personally identifiable information was collected. The study received ethical approval from the Ethics Committee of Anyang Normal University.

To test the proposed hypotheses, this study developed a structural equation model (SEM) from the residents' perspective to examine how city marathons influence tourism development and how residents' attitudes mediate their intentions to support future events. A cross-sectional design was employed (52), and a questionnaire survey was conducted among Kaifeng residents prior to the event to capture their anticipated perceptions of the marathon's potential economic (TEC), image (TIM), and spatial (TSP) impacts. This design allows for a systematic analysis of the structural relationships among residents' perceptions, attitudes, and support intentions at a single point in time, thereby providing an empirical foundation for understanding the socio-psychological mechanisms of sporting events in economically underdeveloped historical and cultural cities.

### 3.1 Questionnaire design

The first section of the questionnaire collected respondents' demographic information. Following the approach of An, Moon, and Norman (53), four basic demographic variables were included: gender, age, education level, and income. The design of the questionnaire items was based on an extensive literature review and subsequently refined through expert evaluation. Five academic experts specializing in tourism management, sports event research, urban development, and sociology were invited to review the questionnaire. They assessed each item in terms of relevance, representativeness, and clarity of expression, providing valuable feedback for revision. Based on their suggestions, several items were refined to ensure better alignment with the study's research objectives and conceptual framework. After revision, the final questionnaire consisted of three main sections, as summarized in Table 1.

**Table 1:** Questionnaire settings.

Section of questionnaire	Dimension	Question design	Main references
Part I: Perceptions of Tourism Impacts	Tourism Economic Impacts (TEC)	The city marathon has attracted a large number of visitors from outside Kaifeng in the short term. (TEC1) The city marathon has boosted tourism consumption in Kaifeng in the short term. (TEC2) The city marathon has extended tourists’ length of stay in the city. (TEC3) The city marathon has increased revenues for tourism-related industries such as transportation, catering, and accommodation. (TEC4) The city marathon has promoted the structural upgrading of Kaifeng's tourism industry. (TEC5) The city marathon has significantly enhanced local residents’ daily consumption levels in Kaifeng. (TEC6)	Zhao et al. (21); Liang et al. (22); Lin et al. (54); Porras-Bueno (55)
	Tourism Image Impacts (TIM)	The city marathon has enhanced Kaifeng's tourism image through online and media publicity. (TIM1) The city marathon has highlighted Kaifeng's image as a historic and cultural city. (TIM2) The city marathon has contributed to improvements in urban infrastructure. (TIM3) The city marathon has promoted the preservation of historical and cultural landmark buildings. (TIM4) The city marathon has improved the quality of public services provided by government institutions. (TIM5) The city marathon has created a unique cultural atmosphere in Kaifeng, strengthening tourists’ cultural identity. (TIM6) The city marathon has enhanced Kaifeng's competitiveness in hosting international sports events. (TIM7)	Gan et al. (56); Du et al. (57); Yamaguchi (58)
	Tourism Spatial Impacts (TSP)	The city marathon has improved connectivity between different cultural attractions within Kaifeng. (TSP1) The city marathon route has become an important sightseeing corridor within the city. (TSP2) The city marathon has provided an opportunity for upgrading Kaifeng's tourism industry. (TSP3) The city marathon has enabled Kaifeng to serve as a hub radiating tourism development to surrounding towns. (TSP4) The city marathon has supported the transformation of Kaifeng's cultural attractions into future tourism hubs. (TSP5)	Wallstam & Kronenberg (59); Smith & McGillivray (60); Lu & Lin (61)
Part II: Residents’ Attitudes	Residents’ Attitudes (RAT)	I feel proud that Kaifeng is hosting the city marathon. (RAT1) I hold a positive attitude toward Kaifeng hosting the city marathon. (RAT2) I believe the city marathon has enhanced Kaifeng's national and international visibility. (RAT3) I believe the city marathon is of great significance to Kaifeng's development. (RAT4) I believe Kaifeng should continue to host the city marathon on a regular basis. (RAT5) I believe the city marathon has strengthened the sense of cohesion among Kaifeng residents. (RAT6) I believe the city marathon has improved my interaction experience with tourists from other cities. (RAT7)	Leoni et al. (62); Yamashita & Hallmann (47); Fam et al. (63); Poczta & Malchrowicz-Mośko (64)
Part III: Support Intention	Residents’ Support Intentions (RIS)	I will continue to support Kaifeng in hosting city marathons in the future. (RIS1) I support Kaifeng in organizing more sports events similar to the city marathon. (RIS2) I am willing to attend the marathon as a spectator. (RIS3) I am willing to serve as a volunteer for the Kaifeng City Marathon. (RIS4)	Park et al. (65); Ajzen (66); Chen et al. (67); Könecke et al. (68)

The first part of the questionnaire focuses on residents' perceptions of the tourism impacts of the city marathon, covering three dimensions: Tourism Economic Impacts (TEC), Tourism Image Impacts (TIM), and Tourism Spatial Impacts (TSP). The questionnaire items were developed based on a review of relevant literature and refined through expert consultation. For the economic impact (TEC) dimension, numerous studies have confirmed the significant contribution of marathon events to local tourism economies (54, 55). Zhao et al. (22) conducted an empirical analysis of marathon events in Shandong Province and found that city marathons can attract a large number of visitors in the short term, generating substantial revenue for related sectors such as transportation, catering, and accommodation. This demonstrates the direct economic stimulus of marathon events to host destinations. Accordingly, this study included items such as “increased number of outside visitors”, “growth in tourism consumption”, and “revenue improvement in related industries”. Moreover, Liang et al. (22), through a meta-analysis grounded in Social Exchange Theory (SET), found that residents' positive perceptions of the economic and social benefits of events are central to forming supportive attitudes. Based on this insight, the present study expanded the economic dimension to incorporate macro-level perceptions such as “employment opportunities” and “industrial restructuring”, aiming to capture a more comprehensive understanding of economic impacts.

Regarding tourism image impacts (TIM), prior research has provided valuable evidence that sports events can reshape how destinations are perceived. Du et al. and Yamaguchi (57, 58) demonstrated that marathons enhance destination image and tourism loyalty by strengthening event brands and promoting cultural experiences. Gan et al. (56) further emphasized that marathon events increase city visibility and enrich the cultural atmosphere through media exposure and symbolic representation. In this study, TIM is therefore defined as residents' perceptions of how the marathon influences Kaifeng's symbolic tourism image and external reputation, such as enhancing its visibility through online and media publicity, highlighting its status as a historic and cultural city, improving the perceived quality of public services, and creating a distinctive cultural atmosphere that reinforces tourists' cultural identity. Accordingly, the items focus on image- and reputation-related outcomes, even when they concern visible improvements in infrastructure or heritage protection, insofar as these changes are interpreted by residents as signals of a stronger tourism image.

For tourism spatial impacts (TSP), existing studies suggest that residents' support for tourism development is largely determined by their perceptions of the spatial environment and the distribution of tourism resources (59–61). These insights resonate with the spatial characteristics of marathon events, which typically make use of city roads and scenic routes as racecourses, connecting cultural attractions and commercial districts and even forming new sightseeing corridors. In contrast to TIM, which concentrates on symbolic and perceptual image-building, TSP in this study is defined as residents' perceptions of changes in the functional organization and connectivity of tourism space, including enhanced linkages between scenic areas, the emergence of sightseeing corridors along the marathon route, and the spatial diffusion of tourism development toward surrounding regions. Consequently, the TSP items emphasize spatial restructuring and tourism resource integration rather than image or branding effects.

The second part of the questionnaire focuses on residents' attitudes toward the city marathon (RAT), comprising six items. Attitude serves as a key psychological mechanism that determines whether residents support the hosting of sporting events, and previous studies offer several relevant insights. Leoni et al. (62), in their investigation of sustainable production practices, found that public attitudes are influenced by an integrated perception of ecological and social benefits. Although their findings originated from agricultural research, the conclusion is equally applicable to the social sustainability of sporting events, suggesting that questionnaire design should include residents' perceptions of the event's social value and public benefits. Yamashita and Hallmann (47), examining the Tokyo Olympic Games, demonstrated that residents' levels of trust and perceived benefits significantly affect their attitudes toward the event. This finding provides theoretical support for including items such as “sense of pride” and “enhancement of city image” to reflect residents' positive emotional responses. Similarly, Fam et al. (63) found that the attractiveness of a marathon destination directly influences participants' intention to engage in the event, indicating that residents' attitudes are closely linked to the overall appeal of the host city. This insight justifies the inclusion of items capturing residents' evaluations of the interconnection between the marathon and city development. Moreover, Poczta and Malchrowicz-Mośko (64), in their case study of the Poznań Half Marathon, emphasized that modern road-running events have transcended their traditional health-related purpose and now function as important vehicles for shaping urban identity and social cohesion. This empirical evidence supports the inclusion of items such as “strengthening community cohesion” in this section. Accordingly, this part of the questionnaire not only measures residents' positive evaluations of the marathon but also captures the event's distinctive role in fostering cultural atmosphere and social integration.

The third section examines residents' intention to support the city marathon (RIS), which consists of four items. Support intention is not only a direct outcome of attitude but also a crucial variable for predicting future behavior. According to Ajzen's Theory of Planned Behavior (66), behavioral intention is the most immediate predictor of actual behavior. This provides a solid theoretical foundation for the “support intention” dimension in this study. Park et al. (65) further demonstrated a positive association between residents' participation in marathon events and their quality of life, indicating that residents' participation experiences can translate into stronger intentions to support future events. This finding validates the inclusion of items such as “continued support for hosting the marathon” in this section. Similarly, Chen et al. (67), in their “One Event, One City” study, revealed that improving event service quality significantly enhances runners' loyalty to the host city, suggesting that residents’ satisfaction with the event may also be linked to their long-term support. Moreover, Könecke et al. (68), from an environmental sustainability perspective, found that marathon participants' willingness to pay was positively aligned with their sense of environmental responsibility and event support. This suggests that residents' support intention is not solely shaped by emotional and economic perceptions but also reflects their identification with the event's sustainability values. Consequently, the items in this section capture the multidimensional drivers of residents' support intentions—encompassing rational behavioral prediction, emotional commitment, loyalty, and a sense of social responsibility.

In summary, by integrating insights from existing empirical studies, the questionnaire items were designed to accurately capture residents' multidimensional perceptions of the tourism impacts of the city marathon. This approach ensures a solid foundation for the subsequent analysis of residents' attitudes and support intentions. All items were measured using a five-point Likert scale ranging from 1 (strongly disagree) to 5 (strongly agree).

### 3.2 Pilot test

Before the formal survey, a pilot test was conducted in December 2024, yielding 128 valid responses. Consistent with the stratified sampling framework used in the main survey, the pilot respondents were recruited proportionally from the five resident groups identified in this study: 35 youth and students, 28 workplace and commercial consumers, 45 community residents, 11 sports enthusiasts, and 9 cultural and tourism practitioners. This composition ensured that the pilot sample reflected the demographic and functional diversity relevant to the study context. The pilot data were then used to assess the construct validity and reliability of the questionnaire. First, five experts in the fields of tourism management, sports event studies, and urban development were invited to evaluate the questionnaire items in terms of relevance, representativeness, and clarity of expression. Based on their feedback, several items were revised before the pilot test.

An exploratory factor analysis (EFA) was then performed on the pilot data (*N* = 128). The results showed a Kaiser–Meyer–Olkin (KMO) value of 0.873 and a significant Bartlett's test of sphericity (*χ*^2^ = 1523.47, df = 325, *p* < 0.001), indicating that the data were suitable for factor analysis. The principal component analysis extracted five latent factors, explaining 67.4% of the total variance, which exceeds the commonly accepted threshold of 60%, suggesting a reasonable factor structure.

Most items exhibited factor loadings above 0.40, but three items showed deficiencies. The item “The city marathon has significantly improved local residents’ daily consumption level (TEC6)” had a loading below 0.40. The item “The city marathon has enhanced Kaifeng's competitiveness in hosting international sports events (TIM7)” exhibited cross-loadings on both economic and image factors. The item “I believe the city marathon has improved my interaction with non-local tourists (RAT7)” also showed low loading and unclear dimensional attribution. Based on these results, these three items were removed. Detailed pilot test data are presented in Supplementary Appendix S1. The remaining items demonstrated good convergent validity within their respective dimensions and were retained in the final questionnaire. The final complete questionnaire is shown in Supplementary Appendix S2.

Regarding reliability, all five latent constructs exhibited Cronbach's *α* coefficients greater than 0.70: Tourism Economic Impacts (TEC) = 0.88, Tourism Image Impacts (TIM) = 0.84, Tourism Spatial Impacts (TSP) = 0.85, Residents' Attitudes (RAT) = 0.93, and Residents' Intention to Support (RIS) = 0.88. These results indicate that the questionnaire possesses high internal consistency. The number of items, Cronbach's α coefficients, and explained variance for each construct are summarized in Table 2.

**Table 2:** Reliability and validity analysis of the questionnaire constructs.

Construct	Number of items	Cronbach's α	Explained variance (%)	Cumulative variance explained (%)
Tourism Economic Impacts (TEC)	6	0.88	20.85	20.85
Tourism Image Impacts (TIM)	7	0.84	16.77	37.62
Tourism Spatial Impacts (TSP)	5	0.85	15.31	52.93
Residents’ Attitude (RAT)	7	0.93	11.98	64.91
Residents’ Intention to Support (RIS)	4	0.88	10.94	75.85

In terms of reliability, the Cronbach's α coefficients of all constructs exceeded the recommended threshold of 0.70, as shown in Table 3. Specifically, the coefficients were 0.88 for Tourism Economic Impacts (TEC), 0.84 for Tourism Image Impacts (TIM), 0.85 for Tourism Spatial Impacts (TSP), 0.93 for Residents' Attitude (RAT), and 0.88 for Residents' Intention to Support (RIS), indicating a high level of internal consistency. Overall, the questionnaire demonstrated satisfactory content validity, construct validity, and reliability, and can therefore be used as a reliable instrument for subsequent empirical analysis.

**Table 3:** Cronbach's α coefficients of each dimension.

Section of questionnaire	Construct	Cronbach's α
Part I: Perceptions of Tourism Impacts	Perceptions of the Economic Impacts of City Marathons on Urban Tourism (TEC)	0.88
	Perceptions of the Image Impacts of City Marathons on Urban Tourism (TIM)	0.84
	Perceptions of the Spatial Impacts of City Marathons on Urban Tourism (TSP)	0.85
Part II: Residents’ Attitudes	Attitudes of Local Residents toward the City Marathon (RAT)	0.93
Part III: Support Intention	Residents’ Intention to Support the City Marathon (RIS)	0.88

### 3.3 Data collection

The questionnaire survey was conducted between January 22 and February 20, 2025, using a combination of on-site distribution and collection methods. The target respondents were local residents of Kaifeng. The survey period took place before the 2025 Kaifeng City Marathon, which was scheduled for March 30, 2025, ensuring that the data captured residents' pre-event perceptions. A team of 20 trained undergraduate students from a Henan-based university was responsible for administering the survey across several major areas of the city.

To ensure sampling consistency and representativeness, the study adopted a stratified quota sampling approach based on population categories, setting survey points within each stratum as follows:

a. Youth and Student Group—This group represents potential volunteers for large-scale sporting events. Survey points were set at Henan University and its surrounding entrances, exits, and public spaces.
b. Workplace and Commercial Consumer Group—As the main commuting and local consumption segment during the event, this group reflects the marathon's impact on daily economic activities and local vitality. Survey points were established in the Gulou–Longting commercial district and the Western Kaifeng business area, covering workplaces and shopping centers.
c. Community Residents Group—Representing the event's influence on local life order and community well-being, this group was surveyed at community service centers and public squares, with one site selected in each direction (east, south, west, and north) to ensure balanced coverage.
d. Sports Enthusiasts Group—As core followers and potential participants or volunteers, this group is more sensitive to the sport–tourism linkage effects. Survey points were located at city sports parks, riverside running tracks, and the main sports complex.
e. Cultural and Tourism Practitioners Group—Directly engaged in tourism service provision, this group captures the event's influence on tourism image building and spatial utilization. Surveys were conducted among local business owners and employees near Qingming Riverside Landscape Garden and the Song Capital Cultural Tourism Zone, explicitly excluding tourists.

This stratified framework, organized by population category, ensured that the most relevant resident groups to the research topic were included. It also enabled quota control and stratified analysis in subsequent statistical procedures.

Before the formal survey, respondents were first informed of the purpose of the study and were asked whether they were familiar with the city marathon event. Only those who answered “familiar” were invited to participate. In total, 937 local residents were approached on-site. Among them, 236 indicated that they were not familiar with the event and were therefore excluded from further participation.

Among them, 236 indicated that they were not familiar with the event and were therefore excluded from further participation. This screening approach aligns with established practices in event-perception research, which typically require a minimum level of awareness to ensure the validity of impact-related assessments. However, this procedure may have inadvertently filtered out residents with lower engagement or weaker event awareness, resulting in a final sample that is relatively more informed—and potentially more supportive—of the event. This limitation is noted and further discussed in relation to the generalizability of the findings.

Of the remaining 701 residents who were familiar with the marathon, 523 valid questionnaires were collected, yielding an effective response rate of 74.6%, as shown in Table 4. The demographic composition of the final sample was as follows. Gender: 52.3% male and 47.7% female. Age: 35.1% were aged 18–25, 34.5% were 26–35, 18.2% were 36–45, 8.7% were 46–55, and 3.5% were above 55 years old. Education level: 44.8% held a bachelor's degree and 16.5% held a postgraduate degree. Occupation: 37.2% were employed in enterprises or institutions, 22.6% were self-employed, and 24.3% were students. Monthly income: 48% earned below RMB 5,000, 33% earned between RMB 5,001 and 10,000, and 19% earned above RMB 10,000.

**Table 4:** Demographic characteristics of respondents.

Sample Item	Category	Number of participants (*N* = 523)	Percentage (%)
Gender	Male	274	52.3
	Female	249	47.7
Age (years)	18–25	184	35.1
	26–35	181	34.5
	36–45	95	18.2
	46–55	46	8.7
	>55	18	3.5
Education Level	Below Bachelor's Degree	205	39.2
	Bachelor's Degree	234	44.8
	Postgraduate Degree	86	16.5
Occupation	Employed (Public/Private Sector)	195	37.2
	Self-Employed	118	22.6
	Student	127	24.3
	Others	83	15.9
Monthly Income (RMB)	<5,000	251	48.0
	5,001–10,000	173	33.0
	>10,000	99	19.0

In addition to these demographic characteristics, the sample distribution across the five resident groups defined in the study's sampling framework was also calculated. Based on the proportional allocation derived from the pilot stage, the final sample included approximately 143 youth and students, 114 workplace and commercial consumers, 184 community residents, 45 sports enthusiasts, and 37 cultural and tourism practitioners. Although these group-level counts are not displayed in Table 4, they correspond to the stratified sampling design and ensure that the survey adequately captures the diversity of the resident population relevant to the research context.

Together, these characteristics indicate that the final sample exhibits a balanced structure and broad coverage of Kaifeng's resident groups, supporting the representativeness and validity of the survey data.

Only respondents who reported being familiar with the city marathon were included in the final analysis. Given the stratified quota design and the screening of “marathon-aware” residents, selection bias cannot be ruled out. We therefore refrain from population-level claims and interpret the findings as analytic generalizations to similar urban contexts.

### 3.4 Data analysis method

To systematically examine the mechanism through which city marathons influence tourism development, data analysis was conducted using SPSS 26.0 (for descriptive statistics and reliability analysis) and AMOS 24.0 (for confirmatory factor analysis and structural equation modeling). Following the two-step approach recommended by Anderson and Gerbing (69), the analysis proceeded as follows. In the first step, confirmatory factor analysis (CFA) was performed to assess the validity and reliability of the measurement model. In the second step, structural equation modeling (SEM) was used to test the hypothesized paths and mediating effects.

This study employed the Maximum Likelihood (ML) method as the primary estimation approach. When the data distribution exhibited significant skewness or the Mardia coefficient indicated a violation of the multivariate normality assumption, the Bollen–Stine Bootstrap or robust standard errors were applied for sensitivity testing to enhance the robustness of model estimation (70, 71).

Before conducting the structural path analysis, it was necessary to ensure that the measurement model met the statistical standards for reliability and validity. Reliability was assessed using internal consistency (Cronbach's α) and composite reliability (CR), both of which are generally required to be ≥0.70 (72, 73). Convergent validity was evaluated through standardized factor loadings and average variance extracted (AVE), with recommended thresholds of ≥0.60 for loadings and ≥0.50 for AVE (74). Discriminant validity was assessed using the Fornell–Larcker criterion (the square root of each construct's AVE should exceed its correlations with other constructs) and further verified using the HTMT ratio (<0.85–0.90) (75). When all these conditions were satisfied, the measurement model was considered to possess acceptable reliability and validity.

To control for common method bias (CMB), both procedural and statistical remedies were applied. Statistically, Harman's single-factor test was conducted to examine whether a single factor accounted for the majority of the covariance among the variables, which would indicate the presence of common method variance. Procedurally, several measures were adopted to minimize respondent consistency bias, including ensuring respondent anonymity, randomizing the order of items, and mixing different scales within the questionnaire. These steps effectively reduced the likelihood of systematic errors caused by common method variance (76). Model fit was evaluated using multiple fit indices. A comparative fit index (CFI) and Tucker–Lewis index (TLI) above 0.90 (preferably ≥ 0.95) indicated good model fit; a root mean square error of approximation (RMSEA) between 0.06 and 0.08 and a standardized root mean square residual (SRMR) below 0.08 were considered acceptable. Additionally, a chi-square to degrees of freedom ratio (*χ*^2^/df) between 1 and 3 suggested satisfactory model fit (77–79). After confirming the adequacy of the measurement model, the structural model was constructed to test the direct and total effects among hypothesized paths. Specifically, the model examined the effects of perceived tourism economic impacts (TEC), tourism image impacts (TIM), and tourism spatial impacts (TSP) on residents’ support intention (RIS), mediated by residents' attitudes (RAT). Furthermore, the coefficient of determination (R²) was reported for endogenous variables to assess the model's explanatory power and theoretical validity (80).

Given that the theoretical model posits residents' attitudes (RAT) as a mediating variable between perceived event impacts and support intention, the present study employed a Bootstrap method with 5,000 resamples to test indirect effects. The bias-corrected (BC) 95% confidence interval (CI) was used to assess the significance of mediation. If the CI for an indirect effect does not include zero, the mediation effect is considered statistically significant.

Following the classification framework proposed by Zhao et al. (81), the detected mediation effects were further categorized as indirect-only mediation, complementary mediation, or competitive mediation, thereby clarifying the underlying mechanism linking residents' perceptions, attitudes, and support intentions. Compared with the traditional Sobel z-test or the Baron–Kenny stepwise approach, the Bootstrap-based estimation provides greater statistical power and more accurate control of Type I error, making it a more robust and appropriate technique for mediation analysis in this study (80, 82).

## 4 Results

### 4.1 Measurement model

To assess the reliability and validity of the measurement instruments, a confirmatory factor analysis (CFA) was conducted on five latent constructs: perceived Tourism Economic Impact (TEC), Tourism Image Impact (TIM), Tourism Spatial Impact (TSP), Residents' Attitude (RAT), and Residents' Intention to Support (RIS). The CFA was followed by estimation of the structural model to ensure the discriminant validity and robustness between the measurement and structural levels. The results are summarized in Tables 5, 6. Using the Maximum Likelihood (ML) estimation method, the overall model demonstrated a good fit to the data: *χ*^2^/df = 1.92, RMSEA = 0.059 (90% CI: 0.052–0.066), CFI = 0.956, TLI = 0.947, and SRMR = 0.046. In addition, NFI = 0.932, GFI = 0.935, and RMR = 0.041, all of which meet the widely accepted thresholds for model fit in the SEM literature (83).

**Table 5:** Reliability and validity of the measurement model.

Construct and Items	Factor loading	*t*-value	α	CR	AVE
Perception of the Economic Impacts of the City Marathon on Urban Tourism (TEC)			0.886	0.892	0.609
The city marathon has attracted a large number of visitors from outside Kaifeng in the short term. (TEC1)	0.78	19.48			
The city marathon has boosted tourism consumption in Kaifeng in the short term. (TEC2)	0.82	20.12			
The city marathon has extended tourists’ length of stay in the city. (TEC3)	0.80	19.80			
The city marathon has increased revenues for tourism-related industries such as transportation, catering, and accommodation. (TEC4)	0.76	19.16			
The city marathon has promoted the structural upgrading of Kaifeng's tourism industry. (TEC5)	0.74	18.84			
Perception of the Image Impacts of the City Marathon on Urban Tourism (TIM)			0.889	0.897	0.574
The city marathon has enhanced Kaifeng's tourism image through online and media publicity. (TIM1)	0.81	19.96			
The city marathon has highlighted Kaifeng's image as a historic and cultural city. (TIM2)	0.79	19.64			
The city marathon has contributed to improvements in urban infrastructure. (TIM3)	0.72	18.52			
The city marathon has promoted the preservation of historical and cultural landmark buildings. (TIM4)	0.75	19.00			
The city marathon has enhanced the quality of public services provided by government institutions. (TIM5)	0.77	19.32			
The city marathon has created a unique cultural atmosphere in Kaifeng, strengthening tourists’ cultural identity. (TIM6)	0.70	18.20			
Perception of the Spatial Impacts of the City Marathon on Urban Tourism (TSP)			0.872	0.879	0.578
The city marathon has improved connectivity between different cultural attractions within Kaifeng. (TSP1)	0.80	19.80			
The city marathon route has become an important sightseeing corridor within the city. (TSP2)	0.76	19.16			
The city marathon has provided an opportunity for upgrading Kaifeng's tourism industry. (TSP3)	0.74	18.84			
The city marathon has enabled Kaifeng to serve as a hub radiating tourism development to surrounding towns. (TSP4)	0.78	19.48			
The city marathon has supported the transformation of Kaifeng's cultural attractions into future tourism hubs. (TSP5)	0.72	18.52			
Residents’ Attitudes toward the City Marathon (RAT)			0.911	0.918	0.631
I feel proud that Kaifeng is hosting the city marathon. (RAT1)	0.83	20.28			
I hold a positive attitude toward Kaifeng hosting the city marathon. (RAT2)	0.85	20.60			
I believe the city marathon has enhanced Kaifeng's national/international visibility. (RAT3)	0.78	19.48			
I believe the city marathon is of great significance to Kaifeng's development. (RAT4)	0.80	19.80			
I believe Kaifeng should continue to host the city marathon on a regular basis. (RAT5)	0.76	19.16			
I believe the city marathon has strengthened the sense of cohesion among Kaifeng residents. (RAT6)	0.74	18.84			
Residents’ Intention to Support the City Marathon (RIS)			0.871	0.877	0.629
I will continue to support Kaifeng in hosting city marathons in the future. (RIS1)	0.84	20.44			
I support Kaifeng in organizing more sporting events similar to the city marathon. (RIS2)	0.81	19.96			
I am willing to attend the marathon as a spectator. (RIS3)	0.77	19.32			
I am willing to serve as a volunteer for the Kaifeng City Marathon. (RIS4)	0.75	19.00			

**Table 6:** Latent correlations and discriminant validity.

Latent Construct	TEC	TIM	TSP	RAT	RIS
TEC	1.00	0.56**	0.52**	0.58**	0.49**
TIM	0.56**	1.00	0.54**	0.61**	0.52**
TSP	0.52**	0.54**	1.00	0.57**	0.50**
RAT	0.58**	0.61**	0.57**	1.00	0.68**
RIS	0.49**	0.52**	0.50**	0.68**	1.00

** (*p* < 0.01).

At the item level, all standardized factor loadings ranged from 0.70 to 0.85 and were statistically significant (*p* < 0.001), indicating that each indicator effectively reflected its latent construct. Consistent with SEM best practices, both Cronbach's α and composite reliability (CR) values exceeded 0.70, with all constructs surpassing the 0.80 benchmark (TEC: α = 0.886, CR = 0.892, AVE = 0.609; TIM: α = 0.889, CR = 0.897, AVE = 0.574; TSP: α = 0.872, CR = 0.879, AVE = 0.578; RAT: α = 0.911, CR = 0.918, AVE = 0.631; RIS: α = 0.871, CR = 0.877, AVE = 0.629).

Convergent validity was confirmed as all AVE values exceeded 0.50, while discriminant validity was supported according to the Fornell–Larcker criterion—the square roots of the AVEs (ranging from 0.758 to 0.795) were greater than all inter-construct correlations.

To further assess potential common method bias (CMB), both statistical and confirmatory tests were performed. A Harman's single-factor test revealed that the first factor explained only 31.4% of the total variance, below the 40% threshold, indicating no severe CMB issue. Furthermore, a CFA single-factor comparison model and a latent method factor (ULMC) sensitivity test were conducted. The single-factor model exhibited significantly poorer fit than the proposed five-factor model (Δ*χ*^2^ = 1,750, Δdf = 5, *p* < 0.001), and the inclusion of a method factor did not meaningfully change the pattern or significance of path coefficients. These results collectively confirmed that common method variance was not a serious concern in this study.

Overall, the measurement model demonstrated satisfactory goodness of fit, internal consistency, convergent validity, and discriminant validity, establishing a robust foundation for subsequent structural analysis.

### 4.2 Structural model

**Figure 1:**
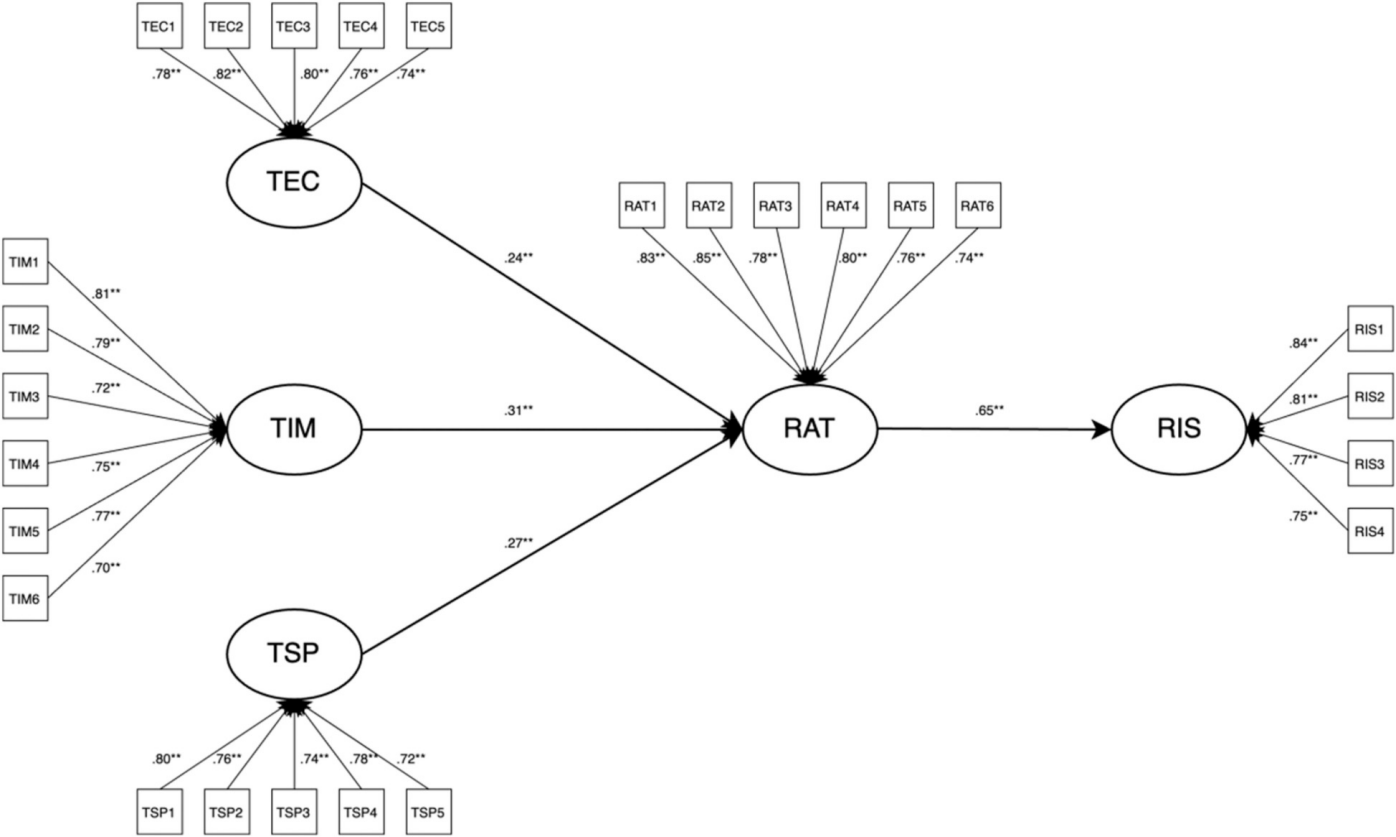
Structural model of residents’ perceptions, attitude, and support intention.

Structural equation modeling (SEM) was employed to examine the relationships among the latent variables. The results of the overall model fit are as follows: *χ*^2^/df = 1.88, RMSEA = 0.057 (90% CI: 0.049–0.064), CFI = 0.958, TLI = 0.950, SRMR = 0.047; NFI = 0.934, GFI = 0.936, and RMR = 0.042. These indices indicate that the hypothesized model fits the observed data well. Table 7 presents the standardized path estimates and their significance levels, while Figure 1 illustrates the structural relationships among the latent constructs.

**Table 7:** Standardized path coefficients, *t*-values, and standard errors.

Path	Standardized coefficient	*t*-value	Standard error	Hypothesis test
TEC → RAT (H1)	0.240	4.800**	0.050	Supported
TIM → RAT (H2)	0.310	6.100**	0.051	Supported
TSP → RAT (H3)	0.270	5.200**	0.052	Supported
RAT → RIS (H4)	0.650	14.200**	0.046	Supported

*Model fit: χ*^2^/df = 1.88, RMSEA = 0.057, CFI = 0.958, TLI = 0.950, SRMR = 0.047. *Explained variance: R*^2^(RAT) = 0.64; *R*^2^(RIS) = 0.56.

** p < 0.01; ns = not significant.

As shown in Table 7, residents' perceptions of the economic (TEC), image (TIM), and spatial (TSP) impacts of the city marathon had significant and positive effects on their attitudes toward the event (RAT) (all *p* < 0.01), with standardized path coefficients of β = 0.24, β = 0.31, and β = 0.27, respectively. Moreover, residents' attitudes exerted a significant positive influence on their future support intention (RIS) (β = 0.65, *p* < 0.01). Overall, all hypotheses (H1–H4) were supported, and the model demonstrated good explanatory power, accounting for 64% of the variance in residents' attitudes and 56% in support intention.

### 4.3 Mediating effects

To examine the mediating role of residents' attitudes (RAT) between the perceived tourism impacts of city marathons and residents' support intentions (RIS), this study incorporated RAT as a mediating variable in the theoretical model to reveal the formation mechanism of residents' behavioral support.

According to Baron and Kenny's framework, three conditions must be met to establish mediation: (1) the independent variable must significantly predict both the mediator and the dependent variable; (2) the mediator must significantly predict the dependent variable; and (3) when both the independent variable and mediator are entered into the model simultaneously, the effect of the independent variable on the dependent variable should become nonsignificant or significantly reduced. If the effect becomes nonsignificant, full mediation is indicated; if it is reduced but remains significant, partial mediation is suggested (80).

Building on these classical conditions, this study further employed the Bootstrap resampling method for statistical inference. By performing 5,000 bootstrap resamples and calculating the bias-corrected (BC) 95% confidence intervals, the significance and stability of the three indirect paths were assessed, yielding more robust estimation results. The outcomes are presented in Table 8.

**Table 8:** Mediating effects of residents’ attitudes (RAT).

Path	Indirect effect	95% BC confidence interval	Direct effect	Total effect	Proportion mediated (%)	Mediation type	Hypothesis test
TEC → RAT → RIS (H5)	0.156	[0.103, 0.217]	0.06 (ns)	0.216	72.2%	Full Mediation	Supported
TIM → RAT → RIS (H6)	0.202	[0.146, 0.271]	0.04 (ns)	0.242	83.5%	Full Mediation	Supported
TSP → RAT → RIS (H7)	0.176	[0.118, 0.239]	0.07*	0.246	71.5%	Partial Mediation	Supported

* *p* < 0.05.

Results show that residents' perceptions of the economic impacts of the marathon (TEC) exert a significant indirect effect on support intention (RIS) through residents' attitudes (RAT) [indirect effect = 0.156, BC 95% CI (0.103, 0.217)]. After including the mediator, the direct effect of TEC on RIS (β = 0.06, t = 1.21, *p* = 0.226) became nonsignificant, indicating full mediation. A similar pattern was observed for the perceived image impacts (TIM), with an indirect effect of 0.202 [BC 95% CI (0.146, 0.271)] and a nonsignificant direct effect (β = 0.04, t = 0.92, *p* = 0.357), suggesting another full mediation. In contrast, the perceived spatial impacts (TSP) demonstrated an indirect effect of 0.176 [BC 95% CI (0.118, 0.239)] and a still significant direct effect (β = 0.07, t = 2.01, *p* = 0.045), implying partial mediation, with 71.5% of its total effect transmitted through residents' attitudes.

In summary, residents' perceptions of the economic (TEC) and image (TIM) impacts of city marathons influence their support intention (RIS) through attitudes (RAT), demonstrating full mediation effects. Meanwhile, spatial perception (TSP) exerts both indirect and direct influences, forming a partial mediation effect. These findings confirm Hypotheses H5–H7 and verify the proposed “Perception–Attitude–Intention” mechanism, which holds true in economically underdeveloped historical and cultural cities such as Kaifeng. Specifically, residents' perceptions of economic and image benefits are primarily translated into support intentions through positive attitudes, while spatial perception continues to exert a direct influence alongside its mediated effect.

## 5 Discussion and conclusion

### 5.1 Key findings

Empirical analysis provides clear evidence of the mechanisms through which residents' perceptions of city marathons influence their attitudes and support intentions. All three perception dimensions—tourism economic impact (TEC; β = 0.24, *p* < 0.01), tourism image impact (TIM; β = 0.31, *p* < 0.01), and tourism spatial impact (TSP; β = 0.27, *p* < 0.01)—exerted significant and positive effects on residents' attitudes toward the marathon (RAT). Among them, the perception of image impact (TIM) had the strongest explanatory power. Furthermore, residents' attitudes showed a strong and positive association with support intentions (RIS) (β = 0.65, *p* < 0.01).

The Bootstrap mediation analysis confirmed that attitude plays a crucial mediating role in transforming perception into behavioral intention. Specifically, attitude acted as a full mediator in the relationships between perceptions of economic and image impacts and support intention, while serving as a partial mediator in the spatial dimension. This suggests that improvements in the city's tourism space not only indirectly foster support through enhanced attitudes but also directly strengthen residents' sense of identification with and endorsement of the event.

These findings lend strong empirical support to the Social Exchange Theory (SET) framework, which posits that individuals' supportive behaviors are shaped by their evaluation of perceived benefits and costs. In line with prior studies, residents who recognize tangible economic and image benefits from marathon events are more likely to develop favorable attitudes, ultimately leading to stronger support intentions (84). This demonstrates that perceived benefits remain the psychological foundation underpinning residents' willingness to support city-level sporting events in economically underdeveloped cultural cities such as Kaifeng.

### 5.2 Research innovations

Grounded in Social Exchange Theory (SET) and the Theory of Reasoned Action (TRA), this study conceptualizes the impacts of city marathons on tourism development across three complementary dimensions: tourism economic impact (TEC), tourism image impact (TIM), and tourism spatial impact (TSP). While the economic and image dimensions have been extensively examined in previous research—demonstrating that sports events stimulate local consumption, revitalize industries, and enhance city visibility and attractiveness through media exposure and brand storytelling (85, 86), systematic investigations into tourism spatial impacts remain scarce. Existing studies have largely focused on isolated cases such as racecourse landscapes, venue construction, or district renewal, without elevating spatial influence to a measurable theoretical construct for quantitative testing. To fill this gap, the present study introduces TSP into a structural equation model and empirically verifies its independent pathway linking residents' attitudes (RAT) and support intentions (RIS). The findings reveal that economic and image perceptions primarily influence residents’ support intentions indirectly through attitudes, whereas spatial perceptions exert both indirect and direct effects. This outcome not only enriches the theoretical framework of tourism impacts generated by city marathons but also highlights the unique value of the spatial dimension within the resident support mechanism.

Furthermore, in contrast to prior research focusing mainly on mega-events and metropolitan contexts (87–89), this study emphasizes the sustainable economic stimulation generated by city marathons in small and medium-sized cities with less-developed economies. Although large-scale events can substantially enhance international visibility and infrastructure in host cities, most existing studies have been concentrated in resource-rich regions, overlooking the developmental needs and latent potential of smaller, less-developed cities. This imbalance may inadvertently exacerbate regional disparities in opportunity access and benefit distribution. Taking Kaifeng, a culturally rich yet economically underdeveloped city, as a case study, the findings demonstrate that residents' perceptions and attitudinal transformations toward marathon events follow the logic of social exchange even under resource constraints. This suggests that residents' support for public events is not solely driven by economic incentives but also shaped by perceived social and cultural returns. Hence, this study extends the explanatory boundaries of Social Exchange Theory in contexts of regional inequality and provides new theoretical insight into understanding resident support mechanisms in less-developed areas.

### 5.3 Practical implications

In terms of event communication and city image building, it is essential to strengthen the cultural core of the city's main narrative. As one of China's eight ancient capitals, Kaifeng's historical heritage and cultural legacy represent its most distinctive resources in marathon promotion. The event can be thematically framed around “Song Dynasty Culture”, linking major landmarks such as Qingming Riverside Landscape Garden, the Ancient City Wall of Bianliang, Longting Lake, Imperial Street, and the Yang Family Mansion to form a symbolic narrative of “Measuring a Millennium City by Running”.

Strategically, a coherent branding system that integrates sports and local culture should be developed. Through cultural co-branding, Song-style visual design, local food festivals, and creative souvenirs, the marathon can be embedded within Kaifeng's broader cultural identity. Such narrative integration enhances residents' sense of place attachment and cultural pride, allowing the city image to emerge through the synergy of sports and heritage, thereby transforming cultural resonance into sustainable resident support.

From a spatial planning perspective, marathon events should be transformed into long-term urban public assets, ensuring that the “post-event legacy” continues to enrich the city's cultural life. The Kaifeng Marathon route can be connected to existing slow-traffic systems, riverside trails, and historical districts—for instance, integrating the Bian River green corridor, the cultural axis from Longting Park to Bao Gong Temple, and the pedestrian system of the Songdu Historical District—to form an open urban network that combines sports, recreation, and cultural functions.

Such spatial reuse enhances residents' everyday experience of the event's legacy and reinforces the inclusiveness of public spaces. The marathon thus evolves from a one-time spectacle into a continuing symbol of cultural memory and urban lifestyle. Through a “post-event, everyday-accessible” spatial legacy, the city can achieve coordinated development across cultural continuity, resident well-being, and tourism vitality.

Finally, from an economic perspective, event organizers should focus on improving residents' perceived benefits to strengthen the motivational link between economic incentives and supportive attitudes. By promoting collaboration between the marathon and local businesses, encouraging partnerships with cultural and culinary brands, hosting community fairs and night-time events, and providing tangible incentives—such as volunteer subsidies, transportation vouchers, or discounted attraction tickets—residents can more directly experience the social and economic returns generated by the event. This transformation—from expected benefit to tangible experience—not only enhances residents' positive attitudes toward the marathon but also fosters a sustainable local support system that integrates community participation, economic vitality, and cultural identity.

### 5.4 Limitations and future research directions

This study, using the Kaifeng City Marathon as a case, demonstrates the applicability of social exchange logic in economically underdeveloped yet culturally rich cities. However, several limitations remain, providing directions for future research.

First, this research adopts a cross-sectional design. Although confirmatory factor analysis and the bootstrap mediation test were employed to enhance model robustness, the design does not completely eliminate potential causal inference bias across time. Future studies could employ longitudinal tracking or quasi-experimental designs to observe changes in residents' perceptions and attitudes before and after marathon events, thereby improving the strength of causal explanations.

Second, the sample data were collected from a single city, which limits the generalizability of the findings across different geographical, economic, and cultural contexts. An additional concern relates to the sampling process. Residents who reported being unfamiliar with the city marathon were excluded to ensure valid responses to perception items. While methodologically reasonable, this approach may have introduced a self-selection bias by disproportionately retaining individuals with higher levels of awareness or prior interest in the event. Consequently, the findings may slightly overestimate supportive attitudes and intentions, and caution is needed when generalizing the results to residents with lower event awareness. Future research could expand to multiple underdeveloped cultural cities, adopt stratified sampling, or include unfamiliar residents as a comparison group to provide a more comprehensive assessment of public attitudes.

Third, the study primarily relies on self-reported measures of residents’ perceptions and attitudes. Although reliability and validity levels meet academic standards, subjective assessments may introduce common method bias. Multiple procedures, including Harman's single-factor test and CFA single-factor comparison, were conducted to minimize this issue; however, the possibility of residual method variance cannot be entirely ruled out.

Finally, this research focuses mainly on the residents' perspective, with limited attention to interactions among other key stakeholders such as government authorities, event organizers, and business entities. Future studies could adopt a multi-stakeholder approach, incorporating constructs like social capital, institutional trust, and organizational collaboration, to build a more holistic framework for understanding how sporting events contribute to sustainable local development.

## Data Availability

The original contributions presented in the study are included in the article/Supplementary Material, further inquiries can be directed to the corresponding author.
