# Supplementary material for: Assessing the tourism impacts of urban marathon events in central China's historic cities: a residents’ SEM analysis
Source: Front Sports Act Living. 2025 Dec 17;7:1720413. doi: 10.3389/fspor.2025.1720413 (PMC12753866; doi:10.3389/fspor.2025.1720413)
Supplement: Supplementary file 1 [file Table1.docx]

## Appendix 1. Exploratory Factor Analysis (EFA) Factor Loadings Results

| **Item Code** | **Item (Description)** | **Economic Impact (TEC)** | **Image Impact (TIM)** | **Spatial Impact (TSP)** | **Resident Attitude (RAT)** | **Support Intention (RIS)** | **Processing Result** |
| --- | --- | --- | --- | --- | --- | --- | --- |
| TEC1 | Attract external visitors | 0.72 | — | — | — | — | Reserved |
| TEC2 | Promote tourism consumption | 0.81 | — | — | — | — | Reserved |
| TEC3 | Extend tourists’ length of stay | 0.76 | — | — | — | — | Reserved |
| TEC4 | Increase revenue from transport, accommodation, and dining | 0.83 | — | — | — | — | Reserved |
| TEC5 | Promote industrial structure adjustment | 0.55 | — | — | — | — | Reserved |
| TEC6 | Enhance local daily consumption | 0.28 | — | — | — | — | Deleted  (Loading < 0.40) |
| TIM1 | Promote city image through media publicity | — | 0.77 | — | — | — | Reserved |
| TIM2 | Highlight cultural city image | — | 0.82 | — | — | — | Reserved |
| TIM3 | Improve infrastructure development | — | 0.68 | — | — | — | Reserved |
| TIM4 | Promote maintenance of cultural heritage sites | — | 0.73 | — | — | — | Reserved |
| TIM5 | Improve quality of public services | — | 0.66 | — | — | — | Reserved |
| TIM6 | Foster cultural atmosphere and identity | — | 0.80 | — | — | — | Reserved |
| TIM7 | Enhance international competitiveness | 0.44 | 0.46 | — | — | — | Deleted (cross-loading > 0.40) |
| TSP1 | Break barriers between scenic spots | — | — | 0.75 | — | — | Reserved |
| TSP2 | Transform marathon routes into sightseeing routes | — | — | 0.82 | — | — | Reserved |
| TSP3 | Drive tourism industry upgrading | — | — | 0.71 | — | — | Reserved |
| TSP4 | Stimulate tourism in surrounding towns | — | — | 0.74 | — | — | Reserved |
| TSP5 | Develop into a tourism hub | — | — | 0.62 | — | — | Reserved |
| RAT1 | Sense of pride in hosting | — | — | — | 0.84 | — | Reserved |
| RAT2 | Overall positive attitude | — | — | — | 0.87 | — | Reserved |
| RAT3 | Enhance city reputation | — | — | — | 0.85 | — | Reserved |
| RAT4 | Perceived significance for development | — | — | — | 0.88 | — | Reserved |
| RAT5 | Advocate long-term hosting | — | — | — | 0.82 | — | Reserved |
| RAT6 | Enhance community cohesion | — | — | — | 0.64 | — | Reserved |
| RAT7 | Improve interaction experience | 0.35 | — | 0.39 | 0.39 | — | Deleted  (low loading and cross-dimensional issue) |
| RIS1 | Continue to support hosting | — | — | — | — | 0.76 | Reserved |
| RIS2 | Support more events | — | — | — | — | 0.82 | Reserved |
| RIS3 | Willingness to attend the event | — | — | — | — | 0.79 | Reserved |
| RIS4 | Willingness to volunteer for the event | — | — | — | — | 0.85 | Reserved |
