# Supplementary material for: Assessing the tourism impacts of urban marathon events in central China's historic cities: a residents’ SEM analysis
Source: Front Sports Act Living. 2025 Dec 17;7:1720413. doi: 10.3389/fspor.2025.1720413 (PMC12753866; doi:10.3389/fspor.2025.1720413)
Supplement: Supplementary file 2 [file Table2.docx]

## Appendix 2. Survey on Residents’ Perceptions of the Kaifeng City Marathon and Its Tourism Impacts

# **Cover Instructions**

Dear Respondent,

This questionnaire is conducted solely for academic research, aiming to examine local residents’ perceptions, attitudes, and support intentions regarding the Kaifeng City Marathon and its tourism impacts. The survey is strictly anonymous and does not collect any personally identifiable information. All responses will be used exclusively for scientific research and statistical analysis, and will not be disclosed to any third party or applied for commercial purposes. Thank you very much for your support and participation!

Please answer according to your genuine feelings. There are no right or wrong answers, and your participation is greatly appreciated.

For each statement, please choose the option that best reflects your opinion:
1 = Strongly Disagree; 2 = Disagree; 3 = Neutral; 4 = Agree; 5 = Strongly Agree

# **Part I: Residents’ Perceptions of the Tourism Impacts of the City Marathon**

## Tourism Economic Impacts (TEC)

| No | Topic | 1 | 2 | 3 | 4 | 5 |
| --- | --- | --- | --- | --- | --- | --- |
| TEC1 | The city marathon has attracted a large number of visitors from outside Kaifeng in the short term. | ☐ | ☐ | ☐ | ☐ | ☐ |
| TEC2 | The city marathon has boosted tourism consumption in Kaifeng in the short term. | ☐ | ☐ | ☐ | ☐ | ☐ |
| TEC3 | The city marathon has extended tourists’ length of stay in the city. | ☐ | ☐ | ☐ | ☐ | ☐ |
| TEC4 | The city marathon has increased revenues for tourism-related industries such as transportation, catering, and accommodation. | ☐ | ☐ | ☐ | ☐ | ☐ |
| TEC5 | The city marathon has promoted the structural upgrading of Kaifeng’s tourism industry. | ☐ | ☐ | ☐ | ☐ | ☐ |

## Tourism Image Impacts (TIM)

| No | Topic | 1 | 2 | 3 | 4 | 5 |
| --- | --- | --- | --- | --- | --- | --- |
| TIM1 | The city marathon has enhanced Kaifeng’s tourism image through online and media publicity. | ☐ | ☐ | ☐ | ☐ | ☐ |
| TIM2 | The city marathon has highlighted Kaifeng’s image as a historic and cultural city. | ☐ | ☐ | ☐ | ☐ | ☐ |
| TIM3 | The city marathon has contributed to improvements in urban infrastructure. | ☐ | ☐ | ☐ | ☐ | ☐ |
| TIM4 | The city marathon has promoted the preservation of historical and cultural landmark buildings. | ☐ | ☐ | ☐ | ☐ | ☐ |
| TIM5 | The city marathon has enhanced the quality of public services provided by government institutions. | ☐ | ☐ | ☐ | ☐ | ☐ |
| TIM6 | The city marathon has created a unique cultural atmosphere in Kaifeng, strengthening tourists’ cultural identity. | ☐ | ☐ | ☐ | ☐ | ☐ |

## Tourism Spatial Impacts (TSP)

| No | Topic | 1 | 2 | 3 | 4 | 5 |
| --- | --- | --- | --- | --- | --- | --- |
| TSP1 | The city marathon has improved connectivity between different cultural attractions within Kaifeng. | ☐ | ☐ | ☐ | ☐ | ☐ |
| TSP2 | The city marathon route has become an important sightseeing corridor within the city. | ☐ | ☐ | ☐ | ☐ | ☐ |
| TSP3 | The city marathon has provided an opportunity for upgrading Kaifeng’s tourism industry. | ☐ | ☐ | ☐ | ☐ | ☐ |
| TSP4 | The city marathon has enabled Kaifeng to serve as a hub radiating tourism development to surrounding towns. | ☐ | ☐ | ☐ | ☐ | ☐ |
| TSP5 | The city marathon has supported the transformation of Kaifeng’s cultural attractions into future tourism hubs. | ☐ | ☐ | ☐ | ☐ | ☐ |

# **Part II: Residents’ Attitudes toward the City Marathon (**RAT)

| No | Topic | 1 | 2 | 3 | 4 | 5 |
| --- | --- | --- | --- | --- | --- | --- |
| RAT1 | I feel proud that Kaifeng is hosting the city marathon. | ☐ | ☐ | ☐ | ☐ | ☐ |
| RAT2 | I hold a positive attitude toward Kaifeng hosting the city marathon. | ☐ | ☐ | ☐ | ☐ | ☐ |
| RAT3 | I believe the city marathon has enhanced Kaifeng’s national/international visibility. | ☐ | ☐ | ☐ | ☐ | ☐ |
| RAT4 | I believe the city marathon is of great significance to Kaifeng’s development. | ☐ | ☐ | ☐ | ☐ | ☐ |
| RAT5 | I believe Kaifeng should continue to host the city marathon on a regular basis. | ☐ | ☐ | ☐ | ☐ | ☐ |
| RAT6 | I believe the city marathon has strengthened the sense of cohesion among Kaifeng residents. | ☐ | ☐ | ☐ | ☐ | ☐ |

## **Part III: Residents’ Intention to Support the City Marathon（RIS）**

| No | Topic | 1 | 2 | 3 | 4 | 5 |
| --- | --- | --- | --- | --- | --- | --- |
| RIS1 | I will continue to support Kaifeng in hosting city marathons in the future. | ☐ | ☐ | ☐ | ☐ | ☐ |
| RIS2 | I support Kaifeng in organizing more sporting events similar to the city marathon. | ☐ | ☐ | ☐ | ☐ | ☐ |
| RIS3 | I am willing to attend the marathon as a spectator. | ☐ | ☐ | ☐ | ☐ | ☐ |
| RIS4 | I am willing to serve as a volunteer for the Kaifeng City Marathon. | ☐ | ☐ | ☐ | ☐ | ☐ |
