# Supplementary material for: Assessing the tourism impacts of urban marathon events in central China's historic cities: a residents’ SEM analysis
Source: Front Sports Act Living. 2025 Dec 17;7:1720413. doi: 10.3389/fspor.2025.1720413 (PMC12753866; doi:10.3389/fspor.2025.1720413)
Supplement: Supplementary file 3 [file Table3.docx]

城市马拉松赛事及旅游影响的问卷调查

# **封面说明**

尊敬的受访者：

您好！本问卷旨在开展学术研究，重点了解当地居民对开封城市马拉松赛事及其旅游影响的感知、态度与支持意愿。问卷采取完全匿名的形式，不涉及任何可识别个人身份的信息，所有数据仅用于科学研究与统计分析，不会对外泄露，也不会用于任何商业目的。请您根据真实感受作答，答案无对错之分。衷心感谢您的支持与参与！

请根据您的真实感受，对以下陈述选择一个最符合的选项：

**1 = 非常不同意；2 = 不同意；3 = 一般；4 = 同意；5 = 非常同意**

# **第一部分：居民对城市马拉松旅游影响的感知（16题）**

## 旅游经济影响（TEC）

| 题号 | 题目 | 1 | 2 | 3 | 4 | 5 |
| --- | --- | --- | --- | --- | --- | --- |
| TEC1 | 城市马拉松短期内吸引了大量外地观众前来开封 | ☐ | ☐ | ☐ | ☐ | ☐ |
| TEC2 | 城市马拉松短期内促进了开封的旅游消费 | ☐ | ☐ | ☐ | ☐ | ☐ |
| TEC3 | 城市马拉松延长了游客在当地的停留时间 | ☐ | ☐ | ☐ | ☐ | ☐ |
| TEC4 | 城市马拉松增加了交通、餐饮和住宿等旅游行业的收入 | ☐ | ☐ | ☐ | ☐ | ☐ |
| TEC5 | 城市马拉松促进了开封旅游产业的结构调整 | ☐ | ☐ | ☐ | ☐ | ☐ |

## 旅游形象影响（TIM）

| 题号 | 题目 | 1 | 2 | 3 | 4 | 5 |
| --- | --- | --- | --- | --- | --- | --- |
| TIM1 | 通过网络媒体等途径宣传开封旅游形象 | ☐ | ☐ | ☐ | ☐ | ☐ |
| TIM2 | 彰显开封历史文化名城的都市形象 | ☐ | ☐ | ☐ | ☐ | ☐ |
| TIM3 | 促进开封基础设施建设 | ☐ | ☐ | ☐ | ☐ | ☐ |
| TIM4 | 促进历史文化标志性建筑物的维护 | ☐ | ☐ | ☐ | ☐ | ☐ |
| TIM5 | 提升了政府机构的公共服务质量 | ☐ | ☐ | ☐ | ☐ | ☐ |
| TIM6 | 城市马拉松营造了开封独特的文化氛围，增强了游客的文化认同感 | ☐ | ☐ | ☐ | ☐ | ☐ |

## 旅游空间影响（TSP）

| 题号 | 题目 | 1 | 2 | 3 | 4 | 5 |
| --- | --- | --- | --- | --- | --- | --- |
| TSP1 | 打通城市内部各个文化景区之间的壁垒 | ☐ | ☐ | ☐ | ☐ | ☐ |
| TSP2 | 促进城市马拉松路线成为城市观光旅游线路 | ☐ | ☐ | ☐ | ☐ | ☐ |
| TSP3 | 为开封旅游业升级提供了一个机遇 | ☐ | ☐ | ☐ | ☐ | ☐ |
| TSP4 | 以开封市为支点辐射周边乡镇旅游业的发展 | ☐ | ☐ | ☐ | ☐ | ☐ |
| TSP5 | 使得开封各个文化景区在未来成为旅游集散地 | ☐ | ☐ | ☐ | ☐ | ☐ |

# **第二部分：居民对城市马拉松赛事的态度（RAT，6题）**

## 居民态度（RAT）

| 题号 | 题目 | 1 | 2 | 3 | 4 | 5 |
| --- | --- | --- | --- | --- | --- | --- |
| RAT1 | 我为开封举办城市马拉松赛事感到自豪 | ☐ | ☐ | ☐ | ☐ | ☐ |
| RAT2 | 我对开封举办城市马拉松赛事持积极态度 | ☐ | ☐ | ☐ | ☐ | ☐ |
| RAT3 | 我认为城市马拉松提升了开封的国际/全国知名度 | ☐ | ☐ | ☐ | ☐ | ☐ |
| RAT4 | 我认为城市马拉松对开封的发展有重要意义 | ☐ | ☐ | ☐ | ☐ | ☐ |
| RAT5 | 我认为开封应该继续长期举办城市马拉松 | ☐ | ☐ | ☐ | ☐ | ☐ |
| RAT6 | 我认为城市马拉松提升了开封居民的凝聚力 | ☐ | ☐ | ☐ | ☐ | ☐ |

# **第三部分：居民支持城市马拉松赛事的意愿（RIS，4题）**

## 居民支持意愿（RIS）

| 题号 | 题目 | 1 | 2 | 3 | 4 | 5 |
| --- | --- | --- | --- | --- | --- | --- |
| RIS1 | 继续支持开封未来举办城市马拉松赛 | ☐ | ☐ | ☐ | ☐ | ☐ |
| RIS2 | 支持开封举办更多像城市马拉松这样的体育赛事 | ☐ | ☐ | ☐ | ☐ | ☐ |
| RIS3 | 愿意到现场观看比赛 | ☐ | ☐ | ☐ | ☐ | ☐ |
| RIS4 | 愿意为开封城市马拉松赛做志愿者 | ☐ | ☐ | ☐ | ☐ | ☐ |
